# Combined treatment of Nimotuzumab and rapamycin is effective against temozolomide-resistant human gliomas regardless of the EGFR mutation status

**DOI:** 10.1186/s12885-015-1191-3

**Published:** 2015-04-11

**Authors:** Dawn Q Chong, Xin Y Toh, Ivy AW Ho, Kian C Sia, Jennifer P Newman, Yulyana Yulyana, Wai-Hoe Ng, Siang H Lai, Mac MF Ho, Nivedh Dinesh, Chee K Tham, Paula YP Lam

**Affiliations:** 1 National Cancer Centre, 11 Hospital Drive, Singapore, 169610 Singapore; 2 National Neuroscience Institute, Singapore, 308433 Singapore; 3 Department of Pathology, Singapore General Hospital, Singapore, 169608 Singapore; 4 Department of Physiology, National University of Singapore, Singapore, 117597 Singapore; 5 Cancer and Stem Cell Biology Program, Duke-NUS Graduate Medical School, Singapore, 169547 Singapore; 6 Division of Neurosurgery, National University Hospital, Singapore, 119074 Singapore

**Keywords:** GBM, EGFR, EGFRvIII, Nimotuzumab, Rapamycin

## Abstract

**Background:**

The treatment of glioblastoma multiforme (GBM) is an unmet clinical need. The 5-year survival rate of patients with GBM is less than 3%. Temozolomide (TMZ) remains the standard first-line treatment regimen for gliomas despite the fact that more than 90% of recurrent gliomas do not respond to TMZ after repeated exposure. We have also independently shown that many of the Asian-derived glioma cell lines and primary cells derived from Singaporean high-grade glioma patients are indeed resistant to TMZ. This issue highlights the need to develop new effective anti-cancer treatment strategies. In a recent study, wild-type epidermal growth factor receptor (wtEGFR) has been shown to phosphorylate a truncated EGFR (known as EGFRvIII), leading to the phosphorylation of STAT proteins and progression in gliomagenesis. Despite the fact that combination of EGFR targeting drugs and rapamycin has been used before, the effect of mono-treatment of Nimotuzumab, rapamycin and combination therapy in human glioma expressing different types of EGFR is not well-studied. Herein, we evaluated the efficacy of dual blockage using monoclonal antibody against EGFR (Nimotuzumab) and an mTOR inhibitor (rapamycin) in Caucasian patient-derived human glioma cell lines, Asian patient-derived human glioma cell lines, primary glioma cells derived from the Mayo GBM xenografts, and primary short-term glioma culture derived from high-grade glioma patients.

**Methods:**

The combination effect of Nimotuzumab and rapamycin was examined in a series of primary human glioma cell lines and glioma cell lines. The cell viability was compared to TMZ treatment alone. Endogenous expressions of EGFR in various GBM cells were determined by western blotting.

**Results:**

The results showed that combination of Nimotuzumab with rapamycin significantly enhanced the therapeutic efficacy of human glioma cells compared to single treatment. More importantly, many of the Asian patient-derived glioma cell lines and primary cells derived from Singaporean high-grade gliomas, which showed resistance to TMZ, were susceptible to the combined treatments.

**Conclusions:**

In conclusion, our results strongly suggest that combination usage of Nimotuzumab and rapamycin exert higher cytotoxic activities than TMZ. Our data suggest that this combination may provide an alternative treatment for TMZ-resistant gliomas regardless of the EGFR status.

## Background

The EGFR signaling system is an attractive target for therapeutic intervention. EGFR gene amplification and overexpression account for approximately 40%-60% of GBMs [1,2]. In a report by Heimberger and colleagues, 42.6% of GBM patients failed to express EGFR, 25.9% had an overexpression of wtEGFR and 31.5% expressed a specific EGFR mutant (EGFRvIII, also known as EGFR type III, de2-7, ΔEGFR) [2]. In a subsequent study, 46% of GBMs lack EGFR [3]. Of note, EGFRvIII expression in GBM is frequently associated with amplification and co-expression of the wtEGFR [4]. Elevated levels of EGFR or EGFRvIII expression confer enhanced cell proliferation and invasion [5]. Given the high frequency of EGFR dysregulation, inhibiting EGFR signaling pathway appears to be a promising and rational therapeutic strategy for attenuating GBM growth.

Nimotuzumab is a humanized monoclonal antibody that targets the extracellular domain of EGFR and inhibits the binding of EGF ligands [6]. It has been extensively used in various solid tumors such as esophageal carcinoma [7], pancreatic cancer [8] and glioma [9,10]. Nimotuzumab has been shown to increase the sensitivity of radioresistant cancer stem cells when used in combination with radiation [11] and prolonged the survival of patients with concurrent radiochemotherapy treatment [12]. Hyperactivation of downstream phosphatidylinositol 3-kinase (PI3K)/AKT/mammalian target of rapamycin (mTOR) pathway is another common occurrence of human glioma [13,14]. mTOR-dependent processes plays a critical role in controlling mRNA translation, ribosome biogenesis, autophagy and metabolism [15]. Rapamycin, also known as Sirolimus, inhibits the highly conserved mTOR by forming a complex with its intracellular receptor, the FK506-binding protein [16]. The latter binds directly to mTOR complex 1 but not mTOR complex 2. Growth factors are known to stimulate mTORC1 through the PI3K/AKT pathway [17]. Rapamycin has been shown to be effective against intracerebral glioma xenografts and has a cytostatic effect against gliomas [18]. It is safe when co-administered with another EGFR tyrosine kinase inhibitor (Gefitinib) in several clinical trials [19,20] and could enhance cell death in combination with Nimotuzumab in epidermoid carcinoma cell line A431 [21].

In a recent study, wild-type epidermal growth factor receptor (wtEGFR) has been shown to phosphorylate a truncated EGFR (known as EGFRvIII), leading to the phosphorylation of STAT proteins and progression in gliomagenesis [22]. This finding may explain earlier report of why EGFRvIII expression rarely occurs without EGFR gene amplification [4]. Despite the fact that combination of EGFR targeting drugs and rapamycin has been used before, the effect of mono-treatment of Nimotuzumab, rapamycin and combination therapy in human glioma expressing different types of EGFR is not well-studied. Herein, we evaluated the efficacy of dual blockage using monoclonal antibody against EGFR (Nimotuzumab) and an mTOR inhibitor (rapamycin) in Caucasian patient-derived human glioma cell lines, Asian patient-derived human glioma cell lines, primary glioma cells derived from the Mayo GBM xenografts, and primary short-term glioma culture derived from high-grade glioma patients. Collectively, our results showed that Nimotuzumab, in combination with rapamycin, significantly enhanced the therapeutic efficacy in human glioma cells compared to single treatment. The combined treatment is effective regardless of the EGFR status in human glioma.

## Methods

### Ethics statement

The use of human glioma cell lines and patient-derived GBMs were approved by the Singhealth Centralized Institutional Review Board, Singapore. GBM tumor specimens from patients, who had undergone surgery, were obtained after written informed consent.

### Drugs and treatment

Temozolomide (TMZ; Temodal) was obtained from Schering Plough and dissolved in DMSO to a final concentration of 100 mM. Rapamycin was obtained from Selleckchem.com (Houston, TX) and dissolved in DMSO to a final concentration of 1 M. Nimotuzumab was provided by Innogene Kalbiotech Pte Ltd (Singapore) at a concentration of 5 mg/ml (equivalent to 0.03 mM). For all treatments, except Nimotuzumab, DMSO was added to a final concentration of 0.1% and used as vehicle control.

### Cell culture

Human glioma Gli36 cells was kindly provided by A.T. Campagnoni (UCLA School of Medicine, Los Angeles, CA) and ΔGli36 cells was provided by M. Sena-Esteves (University of Massachusetts, Boston, MA). Human glioma U87MG was purchased from American Type Culture Collection (Rockville, MD, USA). Human glioma U87MG.EGFRvIII and U87MG.wtEGFR were engineered to express EGFRvIII and wild-type EGFR proteins respectively and were kindly provided by W. Cavenee, Ludwig Institute of Cancer Research, UCSD, CA). Immortalized normal human astrocytes (iNHA) that overexpress E6, E7, and human telomerase reverse transcriptase (hTERT) were kindly provided by R.O. Pieper (University of California, San Francisco, CA) and was cultured in DMEM supplemented with 10% FBS, 0.5 μg/ml puromycin (Invivogen, San Diego, CA), 25 μg/ml blasticidin and 1.25 μg/ml fungizone (Life Technologies, Grand Island, NY). Primary GBM xenograft cell lines, GBM6 and GBM10, were purchased from Mayo Clinic (Rochester, MN) and maintained as subcutaneous xenografts as previously described [23]. Primary Asian glioma cell lines cultivated from Chinese glioma patients, G5T/VGH, GBM8401 and GBM8901 were purchased from Food Industry Research and Development Institute, Bioresource Collection and Research Center (Hsinchu, Taiwan). GBM8401/TSGH, NDMC (abbreviated as GBM8401) was derived from a patient with a right parietal GBM brain tumor [24]. These cells expressed astrocyte-specific intracytoplasmic marker, glial-acidic fibrillary proteins (GFAP), with a doubling time of 38 h and were capable of forming tumors subcutaneously in athymic nude mice. Astrocytic differentiation and growth inhibition could be induced after dibutyryl (db)-cAMP treatment. GBM8901 was also cultivated from a GBM patient. Similar to GBM8401, these cells were GFAP-positive, primarily bipolar and tripolar cells at initial culture but adopted epitheloid-like cell morphology at confluency. Notably, following subcutaneous transplantation, these cells could metastasize to the lung [25]. Lastly, G5T/VGH cells lacked GFAP and only formed tumors initially which regressed gradually.

All cells were maintained at 37°C in a 5% CO2 - 95% air atmosphere and cultured in Dulbecco’s modified Eagle medium (DMEM) supplemented with 10% Fetal Bovine Serum (FBS; Hyclone Laboratories, Logan, UT), penicillin (100 U/ml; Life Technologies, Grand Island, NY), streptomycin (100 μg/ml; Life Technologies) and 2 mM L-glutamine (Life Technologies). ΔGli36 and the U87MG.EGFRvIII cells were further supplemented with 1 μg/ml puromycin (Sigma-Aldrich Corp., St. Louis, MO ) and 500 μg/ml G418 (Life Technologies, Grand Island, NY ), respectively. Primary Asian glioma cell lines GBM8401 and GBM8901 were cultured in RPMI 1640 supplemented with 10% FBS, penicillin/streptomycin and L-glutamine.

### Isolation of primary short-term glioma cultures

High-grade anaplastic astrocytomas, NNI37 and NNI41, were obtained from local patients undergoing tumor resection surgery, following approval of patient informed consent by SingHealth Centralized Institutional Review Board, Singapore. Isolation of cells from patient-derived tumor tissue was performed as follows. In brief, tumor specimens were cut into smaller pieces and washed thoroughly with phosphate-buffered saline (PBS) prior to digestion with 0.25% Trypsin at 37°C for 30 min with constant stirring. Equal volumes of Astrocyte Growth Medium (AGM; Lonza, Basel, Switzerland) were then added to the suspension. Tumor pieces were allowed to settle prior to collecting the supernatant and filtering through a 70-μm membrane filter (BD Biosciences, Franklin Lakes, NJ). Filtered supernatant was centrifuged at 1000 rpm for 5 min at room temperature (r. t). The cell pellet was then resuspended in fresh AGM media and cultured on short-term basis.

### Cell viability assay

Cell viability was determined by cell counting kit-8 (CCK-8) assay (Dojindo, Japan), which measures the number of viable cells based on bioreduction of a water soluble formazan. Human glioma cells (5000 cells/well) were seeded in 96-well dish and 24 h later, Nimotuzumab, rapamycin and respective controls were added to the cells. After an additional 24 h of respective treatments, 10% (v/v) of CCK-8 dye was added into the wells and cells were incubated for 1-2 h. The percentage of viable cells was then determined by measuring absorbance at an optical density (OD) 450 nm with a reference at 650 nm using Victor spectrophotometer (PerkinElmer Life Sciences, Waltham, MA). The percentage cell viability for each cell line and treatment group was normalized to respective vehicle controls.

### Western blot

Equal amounts of proteins were resolved in either 8 or 10% SDS-PAGE and electroblotted onto polyvinylidine difluoride membrane (PVDF) using semi-dry blotting system (Trans-Blot Transfer medium; Bio-Rad Laboratories). Membranes were blocked in 5% BSA in PBS containing 0.1% Tween-20 and incubated overnight at 4°C with antibodies against rabbit anti-phospho-ERK1/2 (Thr202/Tyr204) (1:1000), rabbit anti-ERK1/2 (1:1000), rabbit anti-phospho-AKT (Ser473) (1:500), rabbit anti-AKT (1:1000) from Cell Signaling Technology (Danvers, MA); mouse anti-EGFR (1:200) and anti-pan actin (1:50 000) antibodies from Neomarker (Fremont, CA). After several washes, membrane was incubated with either goat anti-rabbit or goat anti-mouse horseradish peroxidase conjugated secondary antibodies (DakoCytomation, Denmark) (1:10 000). Specific protein bands were visualized with an enhanced chemiluminescence using Western Lightning chemiluminescent kit (Perkin-Elmer, MA).

### Densitometry semi-quantitation analyses of proteins

The band density of specific proteins from each western blot was quantified with MetaVue software (Ver. 6.1) (Molecular Devices Corp.) Briefly, specific protein bands from scanned western films were boxed and integrated intensity values were derived from region measurements. Background was corrected and the intensity of each protein was normalized to the respective loading controls. The activated and total levels of the proteins are expressed as the ratio of intensity of each protein to the respective loading controls.

### Statistical analysis

Statistical differences between values were determined by either one way ANOVA or Student’s *t*-test. A value of *p* < 0.05 was considered as statistically significant. To determine the differences between single and combined treatment’s killing efficacy, one-way ANOVA followed by Tukey’s multiple comparison test (*p* < 0.05) was done using software package Prism 3.0 (Graphpad Software Inc., San Diego, CA).

## Results

### The combination of Nimotuzumab with Sirolimus increased glioma cell cytotoxicity when compared to single drug treatment

**Figure 1:**
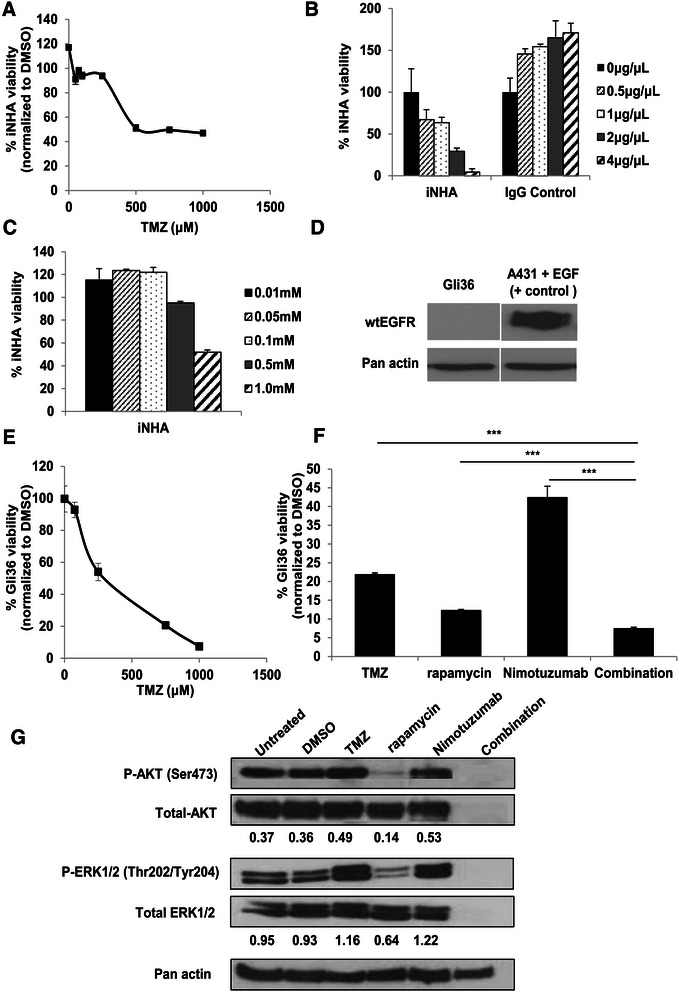
**Enhanced efficacy using rapamycin and Nimotuzumab was observed in EGFR-null cells.** IC50 of **(A)** TMZ, **(B)** Nimotuzumab, and **(C)** rapamycin in iNHA cells was determined by subjecting the cells to a range of drug concentrations. Viability of cells was determined by CCK-8 assay. Data are presented as mean ± SEM. **(D)** Western blot analysis was carried in EGFR-null Gli36 cells to verify the absence of EGFR expression. A431 cells induced with EGF served as positive (+) control for wtEGFR expression. **(E)** IC50 of TMZ was determined in Gli36 cells (from 0 to 1000 μM) as described previously. **(F)** Percentage of cell viabilities of Gli36 cells upon single treatment with either rapamycin (0.1 mM) or Nimotuzumab (0.013 mM) and combination treatment of Nimotuzumab and rapamycin. Data are presented as mean ± SEM. Combination groups were compared to individual single drug treatments ***p < 0.001. **(G)** Western blot analysis of phospho-ERK1/2, total ERK1/2, phospho-AKT and total AKT in Gli36 cells treated with DMSO, TMZ, rapamycin and Nimotuzumab for 24 h. Pan actin served as loading control. The protein expression is normalized to their respective controls in the blots. The numbers below the blot are relative to the respective controls from arbitrary values generated from the MetaVue software, as described in methods.

As TMZ is routinely used as a first-line therapy for GBMs, we sought to first determine the concentration required to achieve 50% inhibition of cell proliferation, i.e., IC50 of TMZ, Nimotuzumab and rapamycin in immortalized human astrocytes (iNHA) which are the most common cell types that give rise to glioma. The results showed that IC50 was achieved at 500 μM of TMZ concentration (Figure 1A). The IC50 for Nimotuzumab was determined to be 2 μg/μl (0.013 mM) (Figure 1B) and these cells exhibited resistance to rapamycin even up to a concentration of 0.5 mM (Figure 1C). However, at this concentration, rapamycin was far too toxic for most of the glioma cells we have tested (data not shown). At a concentration of 0.1 mM, rapamycin exerted differential cytotoxicity levels in iNHA versus human glioma, and thus was chosen for subsequent experiments. Next, we determined the cell kill efficiency of rapamycin and Nimotuzumab in comparison to TMZ in the EGFR-null Gli36 cells, as confirmed by western blot analysis (Figure 1D). TMZ dose response curve indicated that Gli36 cells were sensitive to TMZ treatment with an IC50 of 250 μM (Figure 1E). As shown in Figure 1F, the cell viability in rapamycin and Nimotuzumab treatment groups was approximately 12% and 42% respectively. In combination treatment group, only 7% of the cells were viable.

To gain insight on possible molecular mechanisms involved after respective treatments, western blot analysis was performed to examine the expression levels of activated AKT and ERK1/2, which are downstream targets of EGFR. In these non-EGFR expressing glioma cells, rapamycin treatment reduces activation of AKT at Serine 473 residue when compared to the untreated or DMSO control group (Figure 1G). In contrast, Nimotuzumab treatment did not inhibit pAKT when compared with the control group. Instead, the results showed an increase in activated AKT and ERK1/2 pathways. Despite the detection of similar amount of actin proteins, we are unable to detect the presence of AKT/ERK in combination treatment. In EGF-treated A-431 cells, an epidermoid carcinoma cell line overexpressing wtEGFR, Nimotuzumab did not induce activation of AKT and rapamycin treatment marginally reduces pAKT levels [21]. These data suggested human GBM lacking EGF and its corresponding receptors trigger a non-AKT-dependent pathway in respond to the mono-and combination treatment.

### TMZ-resistant Asian-derived human glioblastoma cell lines were sensitive to co-treatment of Nimotuzumab and rapamycin

**Figure 2:**
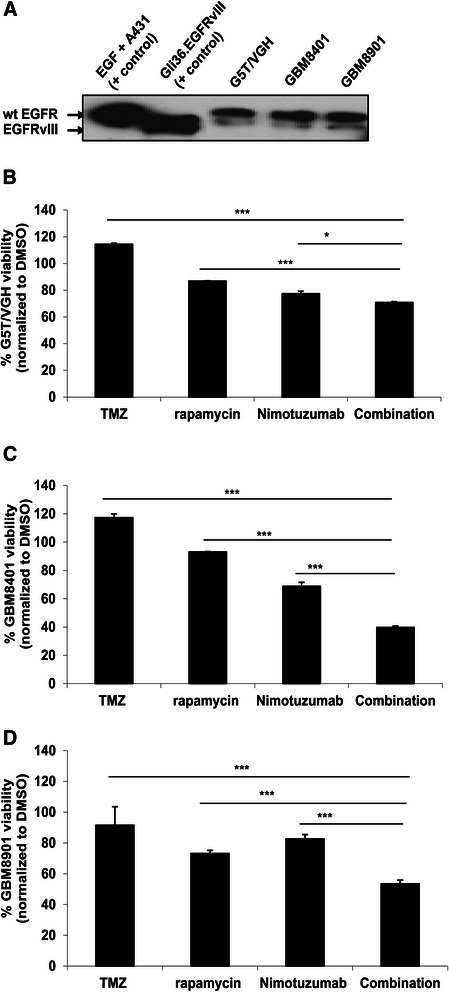
**Nimotuzumab and rapamycin combination treatment enhanced therapeutic efficacy in Asian Glioma cell lines. (A)** Western blot analysis was done to check the EGFR expression status in G5T/VGH, GBM8401 and GBM8901 Asian glioma cell lines with respective (+) controls. These cell lines were treated with TMZ (500 μM), rapamycin (0.1 mM) or Nimotuzumab (0.013 mM) as single treatments or combination of rapamycin and Nimotuzumab for 24 h and percentage of cell viabilities of G5T/VGH **(B),** GBM8401 **(C)** and GBM8901 **(D)** were determined. Data are presented as mean ± SEM. Combination groups were compared to each single drug treatments in individual cell line *p < 0.05, ***p < 0.001.

In Singapore and Asian countries, the molecular signature of gliomas is not well- documented. The latest information available in PubMed is an article dated more than a decade ago by a local neurosurgeon in the “Journal of Neurooncology” where he reported that the genetic profiles of Asian glioma patients do not appear to follow the conventional molecular pathways [26]. Herein, we first determined whether the combination treatment is equally effective in inhibiting tumor cell proliferation as compared to single drug treatment alone in Asian gliomas. All of the Asian patient-derived glioma cells, i.e. GBM8401, G5T/VGH and GBM8901 expressed similar levels of wtEGFR (Figure 2A), and were TMZ-resistant. These cells were only marginally sensitive to Nimotuzumab and rapamycin as a single agent (Figure 2B-D). However, cell viability was reduced 3-fold in GBM8401 and 1.5-fold in GBM8901 with combination treatment in comparison to TMZ (Figure 2C and D). In fact, rapamycin and Nimotuzumab combination treatment significantly reduced the percentage of viable cells by at least 12-50% with respect to the monotherapies (Figure 2; p < 0.01, one-way ANOVA). Taken together, the results showed that combination treatment of Nimotuzumab and rapamycin was more efficacious than TMZ treatment as a single agent.

### The combination of Nimotuzumab with rapamycin enhanced tumor cell cytotoxicity in an EGFR independent manner

**Figure 3:**
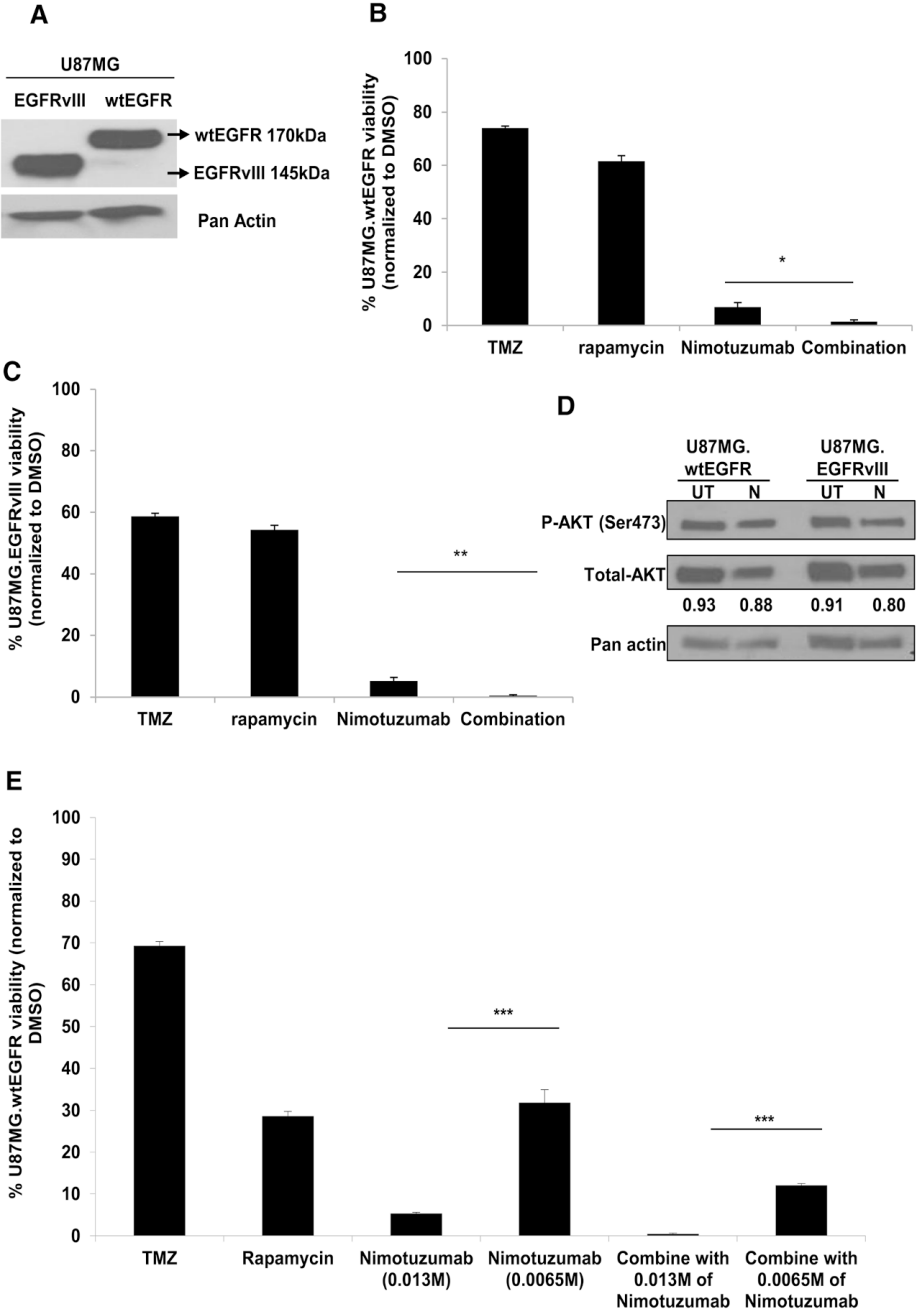
**EGFRvIII conferred enhanced sensitivity to drug treatment in isogenic human glioma cells. (A)** Western blot analysis of EGFR expression in human glioma cells U87MG.EGFRvIII and U87MG.wtEGFR that were engineered to express EGFRvIII and wtEGFR proteins, respectively. Cell viability assay was carried out in **(B)** U87MG.wtEGFR **(C)** U87MG.EGFRvIII cells using CCK-8 assay after treatment with 500 μM of TMZ, 0.1 mM of rapamycin or 0.013 mM of Nimotuzumab and combination of rapamycin and Nimotuzumab. Percentage cell viabilities were measured at 24 h after respective treatments. Data are presented as mean ± SEM. Combination group was compared to Nimotuzumab single treatment for each cell line *p < 0.05, **p < 0.01. **(D)** Western blot analysis was performed to detect the expression levels of phospho-AKT (Ser473) and total AKT in U87MG. wtEGFR and U87MG.EGFRvIII cells untreated (UT) or after treatment with Nimotuzumab (N) for 24 h. Pan actin served as the loading control. Densitometry quantification of the AKT activated levels were determined as described before. The numbers below the blot are displayed as ratio of the total AKT proteins after normalizing with pan actin. **(E)** U87.wtEGFR cells were treated with TMZ (500 μM), rapamycin (0.1 mM) or Nimotuzumab (0.013 mM and 0.0065 mM) as single treatments and combination of rapamycin and Nimotuzumab for 24 h and percentage cell viabilities were determined by CCK-8 assay. Data are presented as mean ± SEM. Nimotuzumab treatment group at half the concentration (0.0065 mM) was compared to the original group (0.013 mM) in single treatments and in combination with rapamycin ***p < 0.001.

EGFRvIII is the most common deletion mutant found in human gliomas [27,28]. However, overexpression of EGFRvIII is usually not observed in isolation, but in combination with amplification of the wild-type receptor, suggesting selective pressure for both species in gliomagenesis [29]. Most recently, wild-type EGFR has been shown to phosphorylate EGFRvIII, leading to phosphorylation of STAT proteins and progression in tumorigenesis [22]. In the earlier part of the study, we showed that combined treatment is effective in Asian glioma cells expressing wild-type EGFR; herein, we sought to determine whether the combination of Nimotuzumab and rapamycin may also be effective in mutant EGFRvIII-expressing human glioma cells. For this, human U87MG glioma cells overexpressing wild-type EGFR (U87MG.wtEGFR; 170 kDa) and mutant EGFRvIII (U87MG.EGFRvIII, 145 kDa), as validated by western blot analysis (Figure 3A), were used. U87MG.wtEGFR and U87MG.EGFRvIII were treated with 500 μM of TMZ, rapamycin, Nimotuzumab and the combination of rapamycin and Nimotuzumab. The enhanced cytotoxic effect in combination treatment was independently confirmed in U87MG.wtEGFR (Figure 3B) and U87MG.EGFRvIII (Figure 3C), respectively. In these glioma cells expressing either wt or EGFRvIII receptor, elevated cell death observed in the Nimotuzumab group correlated with reduced levels of activated AKT in both cell types (Figure 3D), providing further support that Nimotuzumab acts regardless of EGFR status. U87MG.EGFRvIII has lower percentage of viable cells in comparison to U87MG.wtEGFR, suggesting that EGFRvIII-positive human glioma cells were less resistant to TMZ compared to their wtEGFR counterpart. Enhanced cell death in combination treatment versus mono-treatment was also observed in half the normal Nimotuzumab concentration was used in U87MG.wtEGFR (Figure 3E). Similar findings were observed in U87MG.EGFRvIII. Taken together, the data showed that combined treatment is effective regardless of the EGFR status in human glioma.

### Nimotuzumab and rapamycin combination treatment enhanced therapeutic efficacy

**Figure 4:**
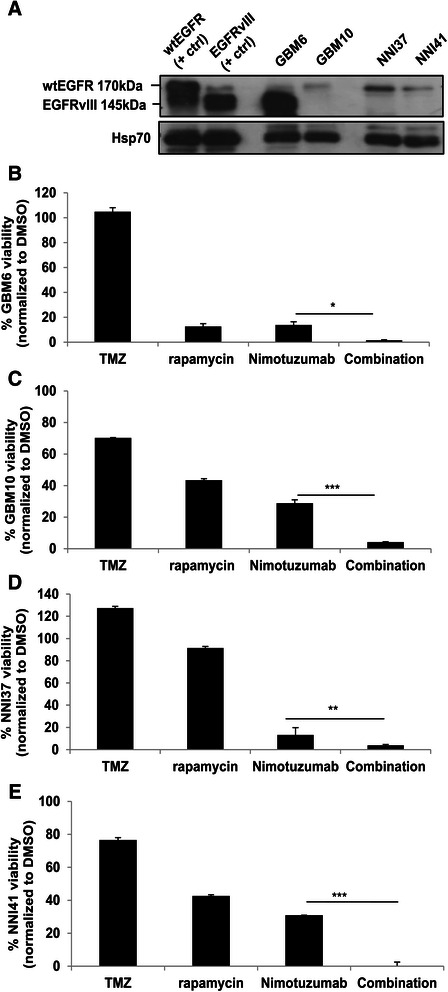
**EGFRvIII-expressing patient-derived glioma cells were more sensitive to Nimotuzumab. (A)** Expression levels of wtEGFR (170 kDa) and EGFRvIII (145 kDa) were analyzed in primary GBM xenografts (GBM6 and 10) and in local patient-derived high grade primary GBMs, NNI37 and 41. Loading controls were performed with Hsp70. The primary GBM xenografts, GBM6 and 10 **(B and C)** and local patient-derived primary GBMs, NNI37 and 41 **(D and E)** were treated with TMZ (500 μM), rapamycin (0.1 mM), Nimotuzumab (0.013 mM) or a combination treatment of Nimotuzumab and rapamycin for 24 h and then cell viabilities were assessed by CCK-8 assay. Data are presented as mean ± SEM. Combination group was compared to Nimotuzumab single treatment for each cell type *p < 0.05, **p < 0.01, ***p < 0.001.

With the aim of translating the combination treatment regimen for clinical use, we studied the effect of combined treatment in patient-derived glioma cells. These tumors were histologically high grade glioma derived from Singaporean patients of Chinese descent and expressed only wtEGFR by western blot analysis (Figure 4A). GBM6 and 10 are primary human GBM xenografts that were originally derived from consented Mayo clinic patients’ tumor specimens. In line with previous studies, our western blot results showed that GBM6 and 10 were EGFRvIII and wild-type EGFR expressing cell lines respectively [30,31]. EGFRvIII-expressing GBM6 was more resistant to TMZ treatment (Figure 4B) when compared to the wtEGFR-expressing GBM10 (Figure 4C). This was not surprising because GBM6 contains unmethylated MGMT promoter [23]. The results showed that although both cell lines were resistant to TMZ treatment, co-treatment of Nimotuzumab and rapamycin was more effective in cell kill when compared to single treatment regardless of the EGFR status of the tumor (Figure 4B and C). These findings were reproducible in wtEGFR expressing high-grade glioma derived from Singaporean patients (Figure 4D and E).

## Discussion

With the increasing ability to dissect cancer genome in clinical tumor samples, instead of having therapies that are based on non-specific one-size-fits-all strategies, moving towards developing new approaches that would allow patient-specific molecular targeted therapies are warranted.

The advances in understanding the molecular basis of cancer formation and progression have unraveled the challenges in treating tumor heterogeneity. It is becoming clear that combination or sequential treatment modalities that target multiple pathways can lead to better control of aberrant cell proliferation. Stupp and colleagues have reported that survival of patients who received the combination therapy of TMZ and radiation exceeded that of radiation alone [32]. Unfortunately, there is no significant benefit of using similar approach in our patient cohort [33]. This may be due to the small number of patient cohort, or perhaps, the genetic aberrations of Asian glioma patients differ from those of Caucasian glioma patients. The astrocytic gliomas derived from Caucasians have been reported to exhibit a different spectrum of genetic abnormalities when compared to non-Caucasian patients [34]. The Cancer Genome Atlas (TCGA) network described a robust gene expression based molecular classification of GBMs that divided them into proneural, neural, classical and mesenchymal subtypes [35]. However, the classical gene signature was not observed in a study conducted by Yan et al. on 225 Chinese glioma patients selected for whole genome gene expression profiling, highlighting differences between Asian gliomas and Caucasian gliomas [36]. Thus, there is a need to identify effective treatment option for Asian glioma patients, and to correlate the outcome to clinical parameters and biomarkers for advancing our understanding of the disease.

In this study, we demonstrate proof of concept that Nimotuzumab and rapamycin is effective as a combination therapy in glioma cell lines derived from Caucasian and Asian glioma patients. The enhanced efficacy of the combination therapy compared to mono-treatment is demonstrated in various human glioma cell lines (Figures 1F, 2B-D, and 3D and E), primary glioma cells derived the Mayo GBM xenografts (Figure 4B and C), and primary short-term glioma culture derived from high-grade Singapore GBM patients (Figure 4D and E). Of note, we are not certain why immortalized normal human astrocytes are not killed by concentrations of TMZ greater than the stated IC_50_ (Figure 1A), it is possible that they have shifted to become more tumor-like cells. These are based on the facts that these cells overexpress viral oncoproteins required for immortalization, and can become tumorigenic in the presence of a Forkhead box transcription factor [37]. For future study, normal primary human astrocytes should be included as additional control. Regardless, it is encouraging that the combination treatment of Nimotuzumab with rapamycin is consistently more effective than the current standard of care therapy, TMZ. One major advantage of Nimotuzumab is that it does not induce skin [38], renal, and gastrointestinal mucosa-related toxicities [39]. Nimotuzumab requires bivalent binding for stable attachment on cell surface receptor while other monoclonal antibody such as Cetuximab can bind in a monovalent manner [40]. Thus, in the skin, where cell surface EGFR density is low, Cetuximab is expected to be more active than Nimotuzumab. It has also been suggested that Nimotuzumab only interferes with ligand-dependent EGFR activation; thus, the basal level of EGFR signalling which is required for the survival of normal epithelial cells is not affected. As a consequence, non-specific toxicity is reduced [6].

The binding affinity and kinetics of Nimotuzumab has been shown to be similar between wtEGFR and EGFRvIII [41]. In non-small cell lung cancer cell lines, the inhibitory effect of Nimotuzumab on EGFR signaling was found to be dependent on the cell surface expression of EGFR but not the status of EGFR mutation [42]. Herein, we showed that Nimotuzumab was effective in Asian patient-derived human glioma cell lines which expressed wild-type EGFR (Figure 2A-D), and Caucasian patient-derived human glioma cell lines expressing either wild-type EGFR (Figure 3B) or mutant EGFRvIII (Figure 3C), indicating that the effect of Nimotuzumab was indeed independent of the endogenous EGFR mutation status [22]. Interestingly, EGFR-null glioma cells (Gli36; Figure 1F) and parental U87MG (data not shown) are also responsive to Nimotuzumab treatment. In Gli36 cells, Nimotuzumab treatment resulted in activation of AKT, thus, the observed reduction in cell viability must be mediated through an AKT-independent pathway (Figure 1G). In U87MG.wtEGFR and U87MG.EGFRvIII cells, Nimotuzumab treatment inhibited activation of AKT, consistent to the reduced pAKT levels in wtEGFR-expressing A431 tumors treated with Nimotuzumab [21]. Although the results indicate that EGFR-null glioma cells are responsive to Nimotuzumab, the mechanism of action is unclear and we cannot exclude the possibility that EGFR-null cells are sensitive to the reagent used to prepare Nimotuzumab, in this case, it is a buffer solution containing polysorbate 80 of unrevealed concentration which has been associated with cytotoxic effect on the cells [43]; the slight reduction in cell viability observed may be attributed to this effect. Alternatively, the findings may represent a new mechanism of action. In normal cells, ligand-stimulated activation of EGFR is followed by subsequent internalization, ubiquitination and degradation in lysosomes. In GBM, mutant EGFRvIII is always present at the cell membrane due to its defective internalization properties [44]. As a result, there is a decrease in associating with Cbl proteins and degradation. The enhanced half-life and the constitutive phosphorylation of EGFRvIII are known to contribute to gliomagenesis. The finding that EGFR-null is also responsive to Nimotuzumab treatment could be due to possible receptor dimerization and crosstalk activities. Receptor dimerization has also been reported between urokinase-type plasminogen activator receptor and EGFRvIII that supports the survival and growth of GBM [45]. Given that receptor dimerization and crosstalk contribute to advantage in cell growth, it is therefore possible that when Nimotuzumab binds to the 3A epitope of the extracellular domain of EGFRvIII, it may be affecting oncogenic receptor dimerization events that lead to reduced cell viability.

The use of rapamycin alone in cancer therapy has shown modest success, perhaps due to the re-assembly of mTOR in the mTORC2/Rictor complex, leading to phosphorylation and reactivation of AKT. James and colleagues have shown that the treatment of A-431 cells with Nimotuzumab is effective in preventing feedback activation of pAKT by rapamycin *in vivo* [21]. Further, tumors derived from combination treatment were compared with mono-therapies using microarray analysis. Combination treatment resulted in the downregulation of genes beyond the typical pathways associated with Nimotuzumab and rapamycin. These pathways include metabolic, ECM-receptor interactions, tight junctions, biosynthesis of unsaturated fatty acids, ubiquitin mediated proteolysis pathways etc. Although this study differs from ours in many ways including experimental objectives, concentration of drugs and presence of EGF ligands and different cancer types, it is nevertheless encouraging that the combination treatment is effective given different cancer model. This is especially relevant in GBM because it highlights the plausibility of targeting TMZ resistant and EGFR-null glioma cells with alternative combination drugs such as Nimotuzumab and rapamycin. Furthermore, Nimotuzumab has recently been shown to enhance cancer radiosensitivity by inhibiting DNA-PKcs activation via the blockage of the PI3K/AKT pathway [46]. Although we have yet to determine whether the radiosensitizing effect of Nimotuzumab may be further enhanced with rapamycin, our results have nevertheless indicated that the combination of Nimotuzumab and rapamycin is more efficacious compared to TMZ and single treatment although it warrants further studies to delineate the underlying mechanism of action given different EGFR receptor status and possible crosstalk interaction.

## Conclusions

The present study showed that the combination of Nimotuzumab and rapamycin could enhance glioma cell death, in an EGFR independent manner. Moreover, the results showed that combination treatment was effective in TMZ-resistant glioma cells, suggesting that Nimotuzumab and rapamycin may potentially be of clinical relevance for future treatment of human gliomas.
